# Intraspecific Variation in Female Sex Pheromone of the Codling Moth *Cydia pomonella*

**DOI:** 10.3390/insects5040705

**Published:** 2014-09-26

**Authors:** Claire Duménil, Gary J. R. Judd, Dolors Bosch, Mario Baldessari, César Gemeno, Astrid T. Groot

**Affiliations:** 1 IBED, University of Amsterdam, Science Park 904, 1098 XH Amsterdam, The Netherlands; E-Mail: cnf.dumenil@gmail.com; 2 Pacific Agri-Food Research Centre, Agriculture and Agri-Food Canada, Box 5000, 4200 Highway 97, Summerland, BC V0H 1Z0, Canada; E-Mail: Gary.Judd@agr.gc.ca; 3 IRTA, Sustainable Plant Protection, Entomology, Avda Alcalde Rovira Roure 191, 25198 Lleida, Spain; E-Mail: dolors.bosch@irta.cat; 4 FEM, Technology Transfer Center, V. E. Mach, 1 38010 S. Michele a/A, Italy; E-Mail: mario.baldessari@fmach.it; 5 Department of Crop and Forest Sciences, University of Lleida, Avda Alcalde Rovira Roure 191, 25198 Lleida, Spain; E-Mail: cesar.gemeno@pvcf.udl.cat; 6 Department of Entomology, Max Planck Institute for Chemical Ecology, Hans Knoell strasse 8, 07745 Jena, Germany

**Keywords:** *Cydia pomonella*, mating disruption, sexual communication, Lepidoptera, Tortricidae, communication interference, codlemone, pheromone

## Abstract

The codling moth, *Cydia pomonella* L. (Lepidoptera, Tortricidae), is a major pest of apple, pear and walnut orchards worldwide. This pest is often controlled using the biologically friendly control method known as pheromone-based mating disruption. Mating disruption likely exerts selection on the sexual communication system of codling moth, as male and female moths will persist in their attempt to meet and mate. Surprisingly little is known on the intraspecific variation of sexual communication in this species. We started an investigation to determine the level of individual variation in the female sex pheromone composition of this moth and whether variation among different populations might be correlated with use of mating disruption against those populations. By extracting pheromone glands of individual females from a laboratory population in Canada and from populations from apple orchards in Spain and Italy, we found significant between- and within-population variation. Comparing females that had been exposed to mating disruption, or not, revealed a significant difference in sex pheromone composition for two of the minor components. Overall, the intraspecific variation observed shows the potential for a shift in female sexual signal when selection pressure is high, as is the case with continuous use of mating disruption.

## 1. Introduction

The evolution of sexual communication in moths is not fully understood, as moth signals and responses are hypothesized to be under stabilizing selection because any deviation away from the mean is selected against [1,2,3]. Communication interference has been found to be a potent environmental variable that can exert strong directional selection on the sex pheromone blend in female moths (e.g., [4,5,6,7,8,9,10]). The presence and abundance of species with similar chemical signals may affect the signal-to-noise ratio (e.g., [11,12,13,14]), which would result in selection for females with the most distinct, optimized pheromone blend (*i.e.*, negative frequency-dependent selection). When specific local environmental conditions persist, selection forces from the environment may result in directional or divergent selection. In the past decade, researchers have described patterns in reproductive traits that are in accordance with such displacement, *i.e.*, greater divergence has been found in mate recognition signals of closely related species in areas of sympatry than in areas of allopatry (e.g., [6,9,10]). It was also shown experimentally that communication interference between the moths *Heliothis virescens* Fabricius and *Heliothis subflexa* Guenée can indeed be a strong directional selection force [7].

The introduction of pheromone-based mating disruption as a large-scale method to control moth pests can be viewed as a large field experiment, on which evolution in action can be assessed. With mating disruption, the air is saturated with sex pheromone of the pest insect, most often using 500–1000 synthetic pheromone dispensers per ha, so that potential mates cannot locate each other (e.g., [15,16]). This communication interference most likely causes strong selection in natural populations to change their sexual communication system [17,18,19], so that shifts in the natural pheromone blend and/or pheromone titers of females or in male response can be expected [20,21,22,23,24,25].

In Japan, the first case of resistance to mating disruption has been described [25]: after 10 years of intense use of mating disruption, a resistant population has developed, in which females have changed their sex pheromone composition and males have widely broadened their pheromone response [22,23,24,25]. This change suggests that moth sexual communication evolves via “asymmetric tracking” [26]: variation in the female pheromone signals can be tracked by males that possess a wide response width, so that a change in the pheromone communication system can occur. A father-son regression in *Trichoplusia ni* Hübner, a pest insect in which a mutant was found in the laboratory rearing, supports the asymmetric tracking hypothesis [27]: males from the normal line showed a broadened response towards the normal and the mutant blend after only three generations of selection.

Mating disruption is used worldwide against the codling moth, *Cydia* pomonella Linnaeus (1758) [16,28]. Codling moths are an economically important pest of pome fruits that requires some level of control [16,19,29,30,31,32,33,34,35]. The pheromone blend of the codling moth has been identified [36] and codlemone (*E,E*)-8,10-dodecadien-1-ol; E8E10-12OH) was found as the major sex pheromone component. Minor pheromone compounds have been identified as well, some of which were found to enhance male antennal response: a saturated alcohol, dodecanol (12OH) [29,37,38,39,40]; two unsaturated alcohols: (*E*)-8-dodecenol (E8-12OH) and (*E*)-9-dodecenol (E9-12OH) [39,40,41], codlemone aldehyde ((*E*,*E*)-8,10-dodecadienal; E8E10-12Al), codlemone acetate ((*E*,*E*)-8,10-dodecadienol acetate; E8E10-12Ac) and four isomers of codlemone: (*Z*,*E*)-8,10-dodecadienol (Z8E10-12OH), (*E*,*Z*)-8,10-dodecadeniol (E8Z10-12OH) and (*Z*,*Z*)-8,10-dodecadeniol (Z8Z10-12OH) [29,40,42,43,44,45,46]. The most widely used mating-disruption formulations against codling moths are codlemone alone or in formulation together with 12OH and 14OH [16,35,47,48,49].

Variation in the sex pheromone composition of *C. pomonella* is largely undocumented. Most studies have analyzed the sex pheromone composition of pooled glands to determine the relative amounts of compounds produced by females, with the aim to identify optimal blends to attract males in wind tunnel assays and field trapping experiments (see Table 1 for an overview of the literature). The only study we know of that has analyzed individual variation in sex pheromone composition of codling moth was conducted by Bäckman *et al.* [50]. The quantities and relative amounts of codlemone, 12OH, 14OH and Z9-12OH in glands, as well as their release rates from the glands, were determined from the last hour of photophase until 3 h into scotophase. This study shows that females started calling one hour into scotophase and that the relative amounts of each compound remained relatively constant after the first hour of calling, and from that point on, the gland content was similar to the volatiles emitted [50]. Such consistency and repeatibility has also been found in other species [51,52,53]. More information concerning any variation in the pheromone blend of *C. pomonella* is not present in the literature.

**Table 1:** Female sex pheromone gland content and male response to pheromone components in *Cydia pomonella* in the literature.

Compound	Gland Content (ng)	Amount (%)	EAG *	Male Attraction ** WT Field		References
(**1**) E8E10-12OH (y)	2.1	100	+++	+	+	[29,37,38,40,42]
(**2**) E8E10-12Ac (n)	0.005	0.01	++	− (>1%) + (<1%)	− (>1%)	[40,43,44,54,55,56]
(**3**) 12OH (y)	1	18.4	+	+	0	[29,37,38,39,40,41,50,57]
(**4**) E8-12OH (y)	nd	0.9	+	0	nd	[40,41]
(**5**) E9-12OH (y)	0.2	5.1	+	0	0	[40,41,42,50]
(**6**) E8E10-12Al (y)	0.02	3.9	+	−	0	[40,41]
(**7**) Z8E10-12OH (y)	0.01	0.8	+	0	nd	[40,43,44,54,55]
(**8**) E8Z10-12OH (na)	0.08	1.8	+	− (>20%)	−	[40,43,44,45,54,55]
(**9**) Z8Z10-12OH (na)	nd	0.3	(+)	− (>20%)	nd	[40,43,44,45,54,55]
(**10**) 10OH (y)	0.005	1.4	0	nd	nd	[29,40]
(**11**) 14OH (n) ^1^	0.2	3.8	0	nd	0	[29,39,40,50]
(**12**) 16OH (na)	0.04	2.6	0	nd	nd	[29,40]
(**13**) 18OH (na)	0.08	3.9	0	nd	nd	[29,40]
(**14**) 18Al (na)	nd	6.3	0	nd	nd	[29,40]
Blend 1 + 3 + 11			nd	+0	+0	[29,38,39,48,58]
Blend 1 + 3 + 5 + 11			nd	nd	0	[50]
Blend 1 + 3 + 4 + 5 + 8 + 11			nd	+	0	[40,56]
Blend 1 + 2 + 8			nd	nd	−	[40]
Blend 1 +3 + 5 + 7 + 8 + 10 + 11 + 12 + 13			nd	0	nd	[59]

(y): Compounds identified and analyzed by GC in this study. (n): Amount too small to be detected in this study. ^1^ Compound could not be detected due to coelution with a non-target compound. (na): Compound not analyzed in this study. * Male response by Electro-antennogram (EAG) to each individual compound, from no response (0) to high response (+++). ** Wind tunnel and field behavioral responses of males to multiple component blends of codlemone (E8E10-12OH) with minor compounds. +: Increase of attraction. −: Decrease of attraction. 0: No difference in attraction, compared to codlemone alone. nd: Not documented.

The aim of our study was to measure intraspecific variation in the sex pheromone content and composition of glands from individual female codling moths. Since mating disruption is an environmentally friendly control method, it would be unfortunate if it loses efficacy due to evolutionary changes in moth populations in response to unchecked pheromone application. To estimate the risk of such evolution occurring, we analyzed the individual sex pheromone glands of females from different origins: one laboratory population from Canada, field populations from Spain, Italy and the Netherlands, where mating disruption has been applied, and a field population from Italy, exposed to insecticides but not to mating disruption.

## 2. Experimental Section

### 2.1. Moths

We compared five populations from various geographic regions that differed in their exposure to mating disruption: a laboratory population, *i.e*., without mating disruption, from Canada, a field population with no mating disruption from Italy, and field populations with mating disruption from Italy, Spain and The Netherlands. We will refer to the populations that have not experienced mating disruption as −MD, and to the populations collected from fields with mating disruption as +MD. In *Cydia pomonella*, late-instar larvae are generally collected from the field by placing cardboard around tree trunks, where the larvae will hide and either enter the pupal stage or diapause. Field collections are thus from larvae that have stopped feeding.

In Canada, pupae were collected from a laboratory colony, which was established in 1993, to support a Sterile Insect Release Program, employed to manage codling moth populations in orchards. The first moths were collected in 1993 in Kelowna, Okanagan Valley, Canada (N49°52'48", W119°26'36.9"), *i.e.*, before mating disruption was employed, and wild males were added to the colony every few years. We refer to this population as Canada −MD.

In Spain, codling moth larvae were collected in July–September 2012 as diapausing larvae from an apple orchard in Lleida (N42°28'23.4", E0°46'17.6"), which has been treated with mating disruption since 2009, using Isomate-C® Plus (Pacific Biocontrol Corporation, Vancouver, WA, USA) that contains three components (Codlemone: 12OH: 14OH in the ratio 100:50:10, respectively). We refer to this population as Spain +MD.

In the Netherlands, late-instar larvae were collected in July and August 2013, from apple orchards in Dreumel (51°50'28.0"N, 5°24'54.6"E), where orchards were treated with mating disruption for at least two years, using Exosex® CM (Exosect, Hampshire, UK) which contains only codlemone. We refer to this population as Netherlands +MD.

In Italy, overwintering larvae were collected in October 2013 from an apple orchard and from a walnut grove. The apple orchard is an experimental plot, 6 years old, located in Castelnuovo, Trento (46°02'48.0"N, 11°28'23.1"E), that has been treated with insecticides only during the last 4 years (Emamectine benzoate—Affirm; Methoxyfenozide—Prodigy; Rynaxypyr—Coragen). We refer to this population as Italy –MD. The walnut grove is 19 years old, it is located in Musile del Piave, Venice (45°35'46.5"N, 12°28'41.5"E), and during the last three years it was treated with mating disruption Puffer® CM (Suterra Europe Biocontrol, Barcelona, Spain), consisting of only codlemone, and additional insecticides (Rynaxypyr—Coragen; Thiaclopryd—Calipso). We refer to this population as Italy +MD.

All larvae and pupae were shipped to the University of Amsterdam, where they were kept individually in a climate room at 23 ± 2 °C, RH 60% ± 10% under a photoperiod L18: D8. Pupae were checked for emergence daily. Emerged females were fed with 10% sucrose until dissection.

### 2.2. Gland Extraction

The glands of 2–4-day-old virgin females were extruded with forceps, 2–3 h after the start of the scotophase. Glands were extracted individually using conical glass vials which contained 50 µL hexane and 200 ng of the internal standard pentadecane. The gland was removed from the solution with forceps after 30 min and the extract was kept at −20 °C until analysis.

### 2.3. Gas Chromatography

The pheromone samples were concentrated under a gentle stream of nitrogen (N_2_) until 2 µL were left. The concentrated pheromone extract was taken up with a 10 µL (701SN Hamilton, Reno, NV, USA) syringe, together with 1 µL of octane to inhibit evaporation, and transferred into a 0.05 mL Micro-insert (Alltech Grom, Rottenburg, Germany) held by a spring fitting into a 1.5 mL vial (Alltech Grom). This vial was then closed with an 11 mm crimp lid, made of aluminum and silicon polytetrafluoroethylene (PTFE) (Alltech Grom). All samples were injected into a gas chromatograph (GC 7890 Agilent Technologies, Santa Clara, CA, USA), equipped with a 7683 automatic injector. The GC was equipped with a high resolution polar capillarity column DB-WAXetr (Agilent Technologies) and a flame ionization detector (FID), and the following temperature program was used: 2 min at 60 °C, increase in temperature of 30 °C per minute, up to 180 °C, followed by a second slower increase (5 °C per min) up to 230 °C. The column was heated to 245 °C for 15 min (20 °C per min) and the FID was kept at 250 °C.

### 2.4. Analyses

All putative pheromone compounds that have been previously identified from the codling moth (see [60]) were purchased from Pherobank (Wageningen, The Netherlands). A multicomponent blend was constructed with all compounds and this was injected into the GC before or after each daily series of injections, to determine the retention times of these compounds and identify them in the gland extract. All GC signals were analyzed with the ChemStation software [61]. Each identified peak was integrated, relative to the amount of internal standard, to calculate the amount of each component in the extract. The relative amounts of the 12 compounds that we could integrate were calculated such that the total amount summed to 100%. Then, we conducted a log contrast transformation by scaling 11 of the 12 compounds that we were able to integrate relative to the 12 h and taking the logarithm of each ratio following Groot *et al*. (2010) [62]. We chose E9-12OH as the divider, because it was the least variable compound in our samples. The pheromone blends of female codling moths were first compared with MANOVA (Wilk’s lambda) to detect overall differences between the sampled populations. Then, each log-transformed component was compared using a single factor ANOVA, followed by a Tukey-Kramer *post-hoc* means separation test. We analyzed differences in relative amounts of the major component (codlemone) and most of the minor compounds: 12OH, E8-12OH, E9-12OH, codlemone aldehyde, codlemone acetate and the stereo-isomer of codlemone (Z8E10-dodecadienol) (see Table 1). We were unable to integrate 14OH (used in mating disruption) due to a contaminant that eluted at the same time in our GC. We were also unable to detect codlemone acetate, which inhibits attraction of males [44,50], most likely due to the fact that we analyzed individual glands. The amount of this acetate has been found to be *ca.* 0.005 ng per gland (0.01% relative to codlemone), and has been detected only in pools of glands [29,40]. We also calculated the ratio of codlemone to dodecanol, because these two compounds have been found to affect male attraction in wind tunnel and/or field experiments (see Table 1). We compared females between regions (Canada, Spain and Italy) and between MD treatments, *i.e.*, +MD and −MD. All statistical analyses were conducted using SPSS 20.0 [63].

## 3. Results

Unfortunately, many females did not emerge or died before pupation, so that our sample sizes were highly variable and sometimes small, especially for females collected as late-instar larvae from mating disruption orchards in Italy (n = 4) and the Netherlands (n = 7). These samples were excluded from the main analysis. However, as this is a first exploratory study, we did include all samples in our first analysis to assess the range of natural variation. The age distribution of the females was similar across populations (*p* = 0.106).

**Figure 1:**
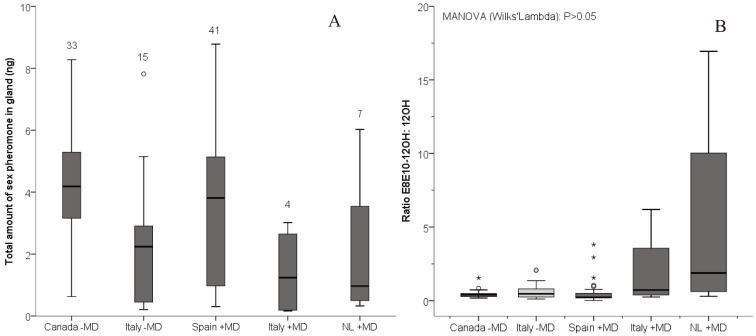
(**A**) Box-whisker plots of the total amount of sex pheromone in individual glands of females from a laboratory population in Canada and field collected females with mating disruption (+MD) in Spain, Italy and The Netherlands, and field collected females without mating disruption (−MD) in Italy. Numbers above the bars are the sample sizes. * *p* < 0.05; (**B**) Box plots showing variation in the ratio of codlemone to dodecanol in each population. See text for further explanation.

Overall, we found a large amount of inter-individual variation among the five groups. Females from Canada contained on average (±SEM) 4.36 ± 0.29 ng of sex pheromone (*i.e.*, all pheromone compounds that we analyzed) in their gland, ranging from 0.62–8.27 ng (see Figure 1A). This was similar to females from Spain (3.64 ± 0.41 ng). Females from the other regions produced somewhat less pheromone, although not significantly, *i.e.*, females from the non-mating disruption field in Italy contained 2.28 ± 0.55 ng, females from Italy +MD contained 1.41 ± 0.72 ng, and females from the Netherlands +MD contained 2.2 ± 0.84 ng. Laboratory-reared females from Canada −MD contained a significantly higher amount of pheromone than field collected females (Spain +MD, Italy +MD and Netherlands +MD) (*p* = 0.008; see Table 2), which is likely due to the fact that the Canadian lab moths are larger than field-collected moths (GJ, personal observation). When we compared the amounts by region, only the total amount of sex pheromone in females from Canada (laboratory) significantly differed from that in females from Italy −MD (*p* = 0.025; see Table 2 and Figure 1A). When comparing the ratio of E8E10-12OH to 12OH, there was no significant difference between any of the populations, most likely due to small sample sizes, but all field populations were highly variable, especially the Netherlands +MD population (Figure 1B).

**Figure 2:**
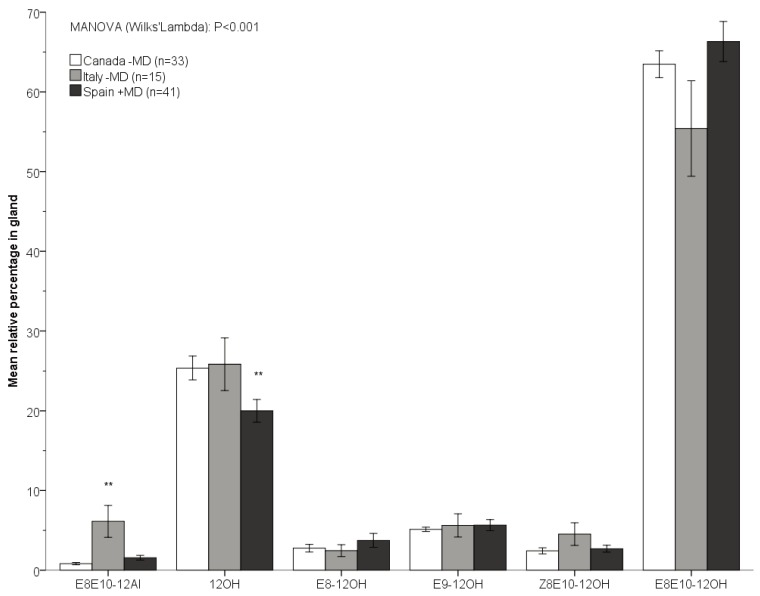
Mean relative percentage (±SEM) of the integrated sex pheromone components in females from the three populations with sample size >10. The sum total of all compounds within a population equals 100%. ** *p* < 0.01. −MD: females collected from a population that had not been exposed to mating disruption; +MD: females collected from a population that had been exposed to mating disruption. See text for further explanation.

When comparing the three populations with sample sizes of n>10 (Spain +MD, Canada −MD and Italy −MD), the overall sex pheromone blend composition was significantly different between codling moth females collected in the field (Spain +MD and Italy −MD) and the laboratory rearing in Canada (MANOVA, Wilk’s Lambda: *p* < 0.001; see Table 2 and Figure 2). Specifically, the relative amount of 12OH (dodecanol) was significantly lower in females from Spain (*p* = 0.002) compared to females from Canada and Italy. The relative amount of codlemone aldehyde (E8E10-12Al) was significantly higher in females from Italy compared to both Canada and Spain (*p* < 0.001). In addition, the overall ratio of codlemone to dodecanol to codlemone aldehyde (E8E10-12OH: 12OH: E8E10-12Al) was significantly different between females from Italy (100:62:18) compared to females from Canada (100:45:2) and Spain (100:50:5) (*p* < 0.001). This was due to the significantly higher proportion of codlemone aldehyde in females from Italy, compared to females from Canada and Spain. When comparing only the females that were collected from the field, excluding the lab-reared population from Canada, females from Spain +MD contained a significantly lower amount of 12OH compared to females from Italy −MD (*p* < 0.001), and the ratio of codlemone to codlemone aldehyde significantly differed between these two populations as well (*p* = 0.001).

**Table 2:** Statistical analysis to compare the sex pheromone amount and composition of *Cydia pomonella* females from different geographic origin, exposed or not to mating disruption.

Source	Sum of Squares	df	Mean Square	*F*-Value	*p*-Value
Dependent variable: Total amount					
Mating disruption (+MD, −MD)	0.102	1	0.102	0.019	0.891
Countries	76.725	4	19.181	3.965	0.005
Canada * Spain +MD					0.632
Canada * Italy −MD					0.025
Canada * Italy +MD					0.093
Canada * Neth +MD					0.135
Spain + MD * Italy −MD					0.250
Spain + MD * Italy +MD					0.307
Spain + MD * Neth +MD					0.499
Neth + MD * Italy −MD					1.000
Neth + MD * Italy +MD					0.979
Italy − MD * Italy +MD					
Dependent variable: Relative amounts					
*Geographic region* (*Canada* −*MD*, *Spain +MD*, *Italy* −*MD*)					
E8E10-12Al	9.787	2	4.893	25.916	<0.001
Canada * Spain					0.148
Canada * Italy					<0.001
Spain * Italy					<0.001
12OH	0.696	2	0.348	4.170	0.018
Canada * Spain					0.373
Canada * Italy					0.112
Spain * Italy					0.007
E8-12OH	0.053	2	0.026	0.093	0.911
Z8E10-12OH	0.916	2	0.458	2.129	0.125
E8E10-12OH	0.146	2	0.073	0.350	0.706
*Exposure* (*+MD* (*Spain*, −*MD* (*Canada and Italy*))					
E8E10-12Al	0.274	1	0.274	0.920	0.340
12OH	0.476	1	0.476	5.603	0.020
E8-12OH	0.161	1	0.161	0.557	0.458
Z8E10-12OH	0.165	1	0.165	0.816	0.369
E8E10-12OH	0.001	1	0.001	0.003	0.958
Dependent variable: Ratio to E8E10-12OH					
*Geographic region* (*Canada* −*MD*, *Spain +MD and Italy* −*MD*)					
12OH	0.507	2	0.254	2.176	0.120
E8E10-12Al	8.019	2	4.009	10.585	<0.001
Canada * Spain					0.488
Canada * Italy					<0.001
Spain * Italy					0.001
*Exposure* (*+MD* (*Spain*), −*MD* (*Canada and Italy*))					
E8E10-12Al	0.249	1	0.249	0.537	0.465
12OH	0.443	1	0.443	3.821	0.054

## 4. Discussion

Overall, we found significant variation in the sex pheromone blend of female codling moths, not only between populations, but also within populations. It is important to note that we only assessed the pheromone composition using pheromone gland extracts, without assessing the pheromone blend emitted from the gland. However, Bäckman *et al.* [50] showed that there is a one to one correlation between the relative amounts of the compounds present in the gland and their emission once the females start calling, *i.e.*, ~2 h into scotophase [50]. Therefore, the variation that we found in the glands likely reflects the variation in pheromone emitted from females. Interestingly, the main variation was not in the major sex pheromone component, codlemone, but in two minor compounds, dodecanol and codlemone aldehyde, the first one of which has been shown to increase attraction of *C. pomonella* males.

The total amount of pheromone produced by a female may depend on her body size [64,65], and may explain that the larger laboratory females from Canada contained more pheromone than field-collected females. Unfortunately, we did not measure the body sizes of the females that we extracted the pheromone glands from. However, even though the total amount of pheromone may be influenced by the body size of the females, this does not seem to affect the composition of the pheromone blends: the pheromone blend from field-collected females in Italy differed more from that of field-collected females from Spain compared to the larger laboratory females from Canada (see Figure 2).

The fact that we found no significant variation in codlemone itself in any comparison suggests that stabilizing selection maintains a relatively constant percentage of this important component. However, a non-significant trend suggests that females collected from a field with mating disruption contained more codlemone than females from a field without mating disruption (see Figure 2). This is in the same direction as was found in the smaller tea tortrix, *Adoxophyes honmai* Yasuda, where females from a population in which resistance to mating disruption occurred also contained significantly more of the major sex pheromone components than females from a susceptible population [23]. Since in both *C. pomonella* and *A. honmai* females perceive their own sex pheromone [66,67,68,69,70], it will be interesting to assess whether females of these two species adjust their pheromone composition depending on the chemical environment, as found previously in *Heliothis subflexa* Guenée [62]. In that case, physiological adjustment may precede genetic differentiation, which may either promote or retard an evolutionary change [71,72].

The pheromone dispensers used in mating disruption in Spain contain codlemone together with dodecanol and tetradecanol, in a ratio of 100:50:20, respectively. The relative amount of dodecanol in females from the field with mating disruption in Spain was significantly lower than in the other females analyzed (Figure 2). However, a similar trend was found in the females from mating disruption fields in Italy and the Netherlands (see Figure 1B), where pheromone dispensers did not contain dodecanol. Hence, while the significant variation in this compound could be due to mating disruption, it might also reflect geographic variation. Codling moths are known to be highly variable genetically between geographical regions. For example, genetic differentiation was found between orchards situated at least 10 km apart, in Europe, South America and South Africa [73,74,75,76,77,78]. A limited gene flow was even found between populations situated less than 1 km apart [73,79,80]. This differentiation is most likely explained by the sedentary behaviour of this moth with a dispersal range of only up to 300 m [79,81]. Such sedentary behaviour increases the chance of population differentiation and possibly also increased variation in sex pheromone communication.

Dodecanol has been found to increase male attraction in some studies [29,40], but not in others [40,48,50,56,58,59], so that its importance in male attraction remains a bit controversial. This may partly explain the significant variation that we found; apparently there is no strong stabilizing selection on the relative amount of this compound. The fact that we found significant variation in another minor compound, codlemone aldehyde, may similarly be explained, as no study so far has shown any relevance of this compound to the attraction of *C. pomonella* males.

Finding a significant amount of variation within and between populations of the codling moth shows the importance of monitoring possible shifts in the sexual communication of this species, including male response. One may expect changes in communications signals when populations have limited gene flow and are exposed to strong selection pressures, such as mating disruption, as was found in the Japanese tea tortrix [24,25]. For a long-lasting, sustainable use of the environmentally friendly control method that mating disruption is, it would be worthwhile to investigate potential additional pheromone blends that can be used alternately with the current pheromone lures, to reduce the selection pressure in a specific direction. A number of studies have already investigated the possible use of kairomones because of their synergism with codlemone and because they are potentially male and female attractants e.g., [67,82,83,84,85,86]. It would be interesting to determine the effect of plant volatiles on female signals to understand how to use them in codling moth management.

In conclusion, we found intraspecific variation in the female sex pheromone of the codling moth, both between females from the same population and between different populations, which could be due to different environmental conditions and/or genetic differentiation. This indicates that the sexual communication system of *C. pomonella* is not stable but subject to variation. Within-species and even within-population variation in the sex pheromone blend has been shown in some other moth species as well [51,68,87,88], suggesting that stabilizing selection may be countered by natural selection, e.g., due to the homing in of natural enemies [89], or due to communication interference with other closely related sympatrically occurring species [6,7,9,10], or due to mating disruption. The general assumption that moth sex pheromones have very low variation because of their importance as species-recognition signals has likely inhibited studies on the extent of intraspecific variation in moth sex pheromone signals. The sedentary behaviour of *C. pomonella* increases the chance of developing resistance against mating disruption, which is thus another reason to monitor possible variation in the sexual communication in populations that are continuously exposed to mating disruption.
